# Evolution of the cardiac dyad

**DOI:** 10.1098/rstb.2021.0329

**Published:** 2022-11-21

**Authors:** John James Mackrill

**Affiliations:** Department of Physiology, School of Medicine, University College Cork, Western Gateway Building, Western Road, Cork T12 XF62, Republic of Ireland

**Keywords:** heart, sarcoplasmic reticulum, t-tubule, dyad, evolution, bridging integrator 1

## Abstract

Cardiac dyads are the site of communication between the sarcoplasmic reticulum (SR) and infoldings of the sarcolemma called transverse-tubules (TT). During heart excitation–contraction coupling, Ca^2+^-influx through L-type Ca^2+^ channels in the TT is amplified by release of Ca^2+^-from the SR via type 2 ryanodine receptors, activating the contractile apparatus. Key proteins involved in cardiac dyad function are bridging integrator 1 (BIN1), junctophilin 2 and caveolin 3. The work presented here aims to reconstruct the evolutionary history of the cardiac dyad, by surveying the scientific literature for ultrastructural evidence of these junctions across all animal taxa; phylogenetically reconstructing the evolutionary history of BIN1; and by comparing peptide motifs involved in TT formation by this protein across metazoans. Key findings are that cardiac dyads have been identified in mammals, arthropods and molluscs, but not in other animals. Vertebrate BIN1 does not group with members of this protein family from other taxa, suggesting that invertebrate BINs are paralogues rather orthologues of this gene. Comparisons of BIN1 peptide sequences of mammals with those of other vertebrates reveals novel features that might contribute to TT and dyad formation. The analyses presented here suggest that the cardiac dyad evolved independently several times during metazoan evolution: an unexpected observation given the diversity of heart structure and function between different animal taxa.

This article is part of the theme issue ‘The cardiomyocyte: new revelations on the interplay between architecture and function in growth, health, and disease’.

## 1. Introduction

There is uncertainty as to when, in evolutionary time, that hearts first appeared [1]. This is confounded by the definition of the term ‘heart’, which between different taxonomic groups, varies in terms of its developmental origin; the nature of the force-generating cells; whether it drives an open or closed circulation; dorsal or ventral location in the body; and the number of chambers present [2]. However, based on the anatomy, physiology and molecular biology of extant groups, the development of true hearts probably required the emergence of a triploblastic, bilaterian (bilaterally symmetrical) body-plan (table 1).

**Table 1.** Summary of detection of cardiac dyads in different taxa. (Listed are the different taxa, the presence of striated cardiomyocytes, the detection of TT or dyads and the citation sources.)

taxonomic rank 1	taxonomic rank 2	species	striated cardiomyocytes	TT/dyads reported	citation(s)
Porifera	Demospongiae	*Geodia cydonium*	no	no	[3]
Ctenophora	Nuda	*Beroe ovata*	no	no	[4]
Ecdysozoa	Arthropoda	multiple	yes	yes	[5–12]
Ecdysozoa	Nematoda	*Caenorhabditis elegans*	yes	no	[13]
Lophotrochozoa	Mollusca	multiple	yes	in some	[14–17]
Lophotrochozoa	Annelida	multiple	yes	no	[13,18–21]
Ambulacraria	Echinodermata	*Parastichopus tremulus*	no	no	[22]
Chordata	Tunicata	*Corella willmeriana*	no	no	[23]
Chordata	Myxiniformes	*Myxine glutinosa*	yes	no	[24]
Chordata	Petromyzontiformes	*Lampetra fluviatilis*	yes	no	[25]
Chordata	Chondrichthyes	several	yes	no	[26]
Chordata	Actinopterygii	multiple	yes	no	[27–34]
Chordata	Amphibian	*Rana pipiens*	yes	no	[35]
Chordata	Reptilia (including Aves)	multiple	yes	possibly short TT in *Amyda* sp.	[36–40]
Chordata	Mammalia	multiple	yes	yes	[41–49]

The current review will focus on the evolution of a subcellular domain involved in efficient excitation–contraction coupling (ECC) in mammalian cardiomyocytes: the dyad junction. This will involve examination of ultrastructural and molecular evidence for the presence of these membrane contact sites among distinct metazoan (animal) groups. This information will be used to gain insights into the evolution of cardiac dyad junctions.

## 2. Ultrastructural hallmarks of the cardiac dyad

**Figure 1.**
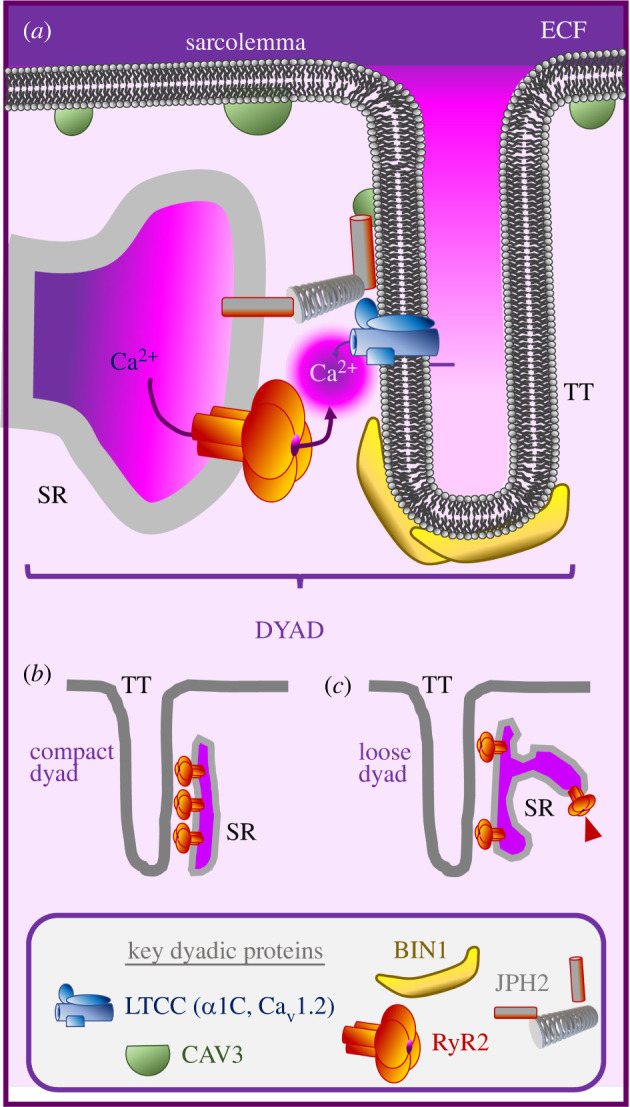
Molecular features of the mammalian cardiac dyad. (*a*) At the dyad junction, transverse-tubular (TT) depolarization is sensed by the L-type Ca^2+^ channel (LTCC, containing the cardiac Ca_v_1.2 (α1C) subunit). This opens an intrinsic Ca^2+^ channel, allowing influx of Ca^2+^ from the extracellular fluid (ECF) into the dyadic cleft, gating the type 2 ryanodine receptor (RyR2) in a Ca^2+^-induced Ca^2+^-release process. Key proteins involved in the formation and function of the cardiac dyads are the bridging integrator 1 (BIN1), junctophilin 2 (JPH2) and caveolin 3 (CAV3). Panel (*b*) illustrates the features of ‘compact dyads’: the flattened cisternae of the sarcoplasmic reticulum (SR) are completely aligned with the TT; RyRs are only located within the junctions between these membranes. In the case of ‘loose’ dyads, panel (*c*), the terminal SR extends into the sarcoplasm and contains extra-junctional RyRs (red arrowhead).

The evolution and comparative physiology of cardiac ECC has been extensively summarized in previous reviews [50,51]. The work presented here focuses on investigating evolution of the cardiac dyad, a key membrane contact site involved in the ECC of mammalian hearts, figure 1. This will be achieved by surveying published ultrastructural evidence for the presence of transverse-tubules (TT) and dyads across metazoan (animal) taxa; by reconstructing the evolution of key proteins involved in formation of these structures within these groups; and by analysing features of these proteins that might contribute to cardiac dyad form and function.

### (a) Mammals

As described in detail in other articles in this *special issue*, dyad junctions of mammalian cardiomyocytes are the interface between TT of the sarcolemma and terminal cisternae of the sarcoplasmic reticulum (SR). These membrane contact sites contain voltage-sensing L-type Ca^2+^-channels (LTCC; containing the α1C subunit of the dihydropyridine receptor, also called Ca_v_1.2) in the TT and type 2 ryanodine receptor Ca^2+^-release channels (RyR2) in the SR, together forming Ca^2+^-release units (CRUs). During ECC, sarcolemmal depolarization causes a conformational change in the LTCC, gating its channel and allowing Ca^2+^-influx, which is amplified by opening of RyR2, in a process termed Ca^2+^-induced Ca^2+^-release (CICR), figure 1. Ca^2+^-release overcomes buffering to allow the Ca^2+^ to spread from the dyadic nanodomains to the bulk sarcoplasm, activating the contractile apparatus, leading to force production. Similar junctions are present in mammalian skeletal muscle but are triadic (two TC-SR contacts per TT); possess a distinct type of LTCC (α1S or Ca_v_1.1); and Ca^2+^-release channel (RyR1); and operate by a conformational coupling mechanism, rather than by CICR. Within mammalian cardiomyocytes, peripheral couplings between the sarcolemma and SR also contain CRUs and contribute to ECC. The mechanism of dyad formation is uncertain, but probably involves invagination of the sarcolemmal and recruitment of addition TT membranes, either pre-coupled to the SR or delivered independently of it [52]. In *Danio rerio* (zebrafish) skeletal muscle, genetic manipulations, microscopy and mathematical modelling indicate that TT are formed by an endocytic capture mechanism [53].

The ultrastructure of the mammalian TT system varies between species [41]; during development [42,43,52]; between different heart chambers [54] and in the aetiology of myocardial disease [55,56]. This system consists of TTs that are aligned with sarcomeric Z-discs, interconnecting with longitudinal TTs, which run parallel with the long axis of the cardiomyocyte [57]. Atrial cardiomyocytes have a less developed, and in some cases absent, TT system compared with those of the ventricle [54]. Dyads are sparse or undetectable in Purkinje fibres of the conduction system. Cardiac CRUs are also formed at peripheral couplings between the sarcolemma and TC-SR [58]. Cardiac dyads are diverse in terms of their size, morphology and localization. A recent ultrastructural study of rat ventricular myocytes defined two main classes of dyad termed ‘compact’ (tight association between TC and TT) and ‘loose’ (with wider dyadic clefts). In an isoproterenol-induced model of myocardial injury, the redistribution of dyadic populations from compact to loose types mirrored the decrease in efficiency of ECC [59].

Ultrastructural evidence for the presence of cardiac dyads has been reported in most orders of mammals. Among eutherian mammals, these include rodents (e.g. mice, *Mus musculus,* [60]); bats (e.g. the West African bat, *Eidolon helvum*, [61]); lagomorphs (e.g. the rabbit *Oryctolagus cuniculus*, [60]); cetaceans (e.g. the bowhead whale, *Balaena mysticetus*, [44]); proboscids (e.g. the African elephant, *Elephas maximus*, [45]); carnivores (e.g. the domestic cat, *Felis catus* [42] and dog *Canis lupus familiaris*, [46]); artiodactylids (e.g. the pig, *Sus scrofa*, [54]) and perissodactylids (e.g. the horse, *Equus caballus*, [41]). The presence of cardiac dyads has not been reported in insectivorans (shrews, moles, desmans and others), pinnipeds (walruses, seals and sea-lions), sirenids (manatees) or tubulidents (aardvarks). However, functional evidence supports that SR Ca^2+^ release of large magnitude is triggered by a small influx of through LTCCs in the ventricles of the common shrew, *Sorex araneus* [47] supporting a CICR mechanism of ECC. Among non-eutherian mammals, TTs and dyads have been characterized during the development of the right ventricle of the opossum *Didelphis virginiana*, a marsupial. In *Di. virginiana*, it was noted that TT were first detectable at postnatal day 43 and was fully developed by day 105 (fully weaned joeys) [43]. To date, no published information is available on the myocardial ultrastructure of monotremes, such as *Platypus* and *Echidna*.

### (b) Non-mammalian vertebrates

Neither TT nor dyads have been detected in the heart tissues of diverse avian species, including in pigeon (*Columba livia*) [48]; the rufous-tailed hummingbird (*Amazilia tzacatl*) [49]; the chicken (*Gallas gallas*) and the zebrafinch [36]. In terms the kinetics of cardiac ECC, birds have overcome the limitations imposed by Ca^2+^ buffering by several mechanisms [50]. Avian cardiomyocytes are long and narrow in diameter compared with their mammalian counterparts, meaning that Ca^2+^ entering via LTCCs and released from peripheral SR only needs to diffuse short distances to activate the contractile machinery. The ventricular myocytes of zebrafinch and chicken possess extensive peripheral SR and extra-junctional or corbular SR, enriched in RyR channels [48]. Corbular SR serves to transmit Ca^2+^ signals deep within these cells via a CICR mechanism [36]. The Ca^2+^ accumulating capabilities of SR from the atria and ventricles of Japanese quail (*Coturnix japonica*) exceed those of their mammalian counterparts, supporting this CICR ‘relay station’ role [49], which facilitates the superior force generation in birds relative to other vertebrates.

Fossil evidence indicates that birds diverged from other theropod dinosaurs during the Jurassic Period, 165–150 Myr [37]. Unlike endothermic birds, most extant reptiles are ectothermic. Morphologically, reptilian cardiomyocytes resemble those of birds: they are long and thin relative to those of mammals. Unlike birds and mammals, no evidence of corbular SR has been reported in non-avian reptile myocardia [49]. There is conflicting evidence regarding the presence of TT and dyads in reptiles. No TTs were detected in the red-eared terrapin (*Trachemys scripta elegans*) [38], in snakes [62], nor in the savannah monitor lizard (*Varanus exanthematicus*) [63]. However, an ultrastructural study indicated the presence of ‘short TT’ (TT of limited extension into the cardiomyocyte) in the myocardium of the soft-shelled turtle *Amyda* sp*.* [64]. Whether this represents a technical artefact or a genuine disparity between reptilian taxa, merits further investigation. In reptiles with high-performance hearts (capable of high cardiac outputs and the development of high blood-pressures), such as *V. exanthematicus* normal ECC appears to occur predominantly through Ca^2+^ influx through the LTCCs. During times of greater demand, such as stimulation by cathecholamines, increased Ca^2+^ influx through the reverse mode of sodium–calcium exchangers (NCX) and enhanced CICR underpin enhanced ECC [63].

Despite their importance in the history of scientific discovery of ECC in skeletal muscle, there are few reports describing the ultrastructure of the myocardium in amphibians. Ventricular myocytes from the northern leopard frog, *Rana pipiens*, are very narrow (less than 5 μm in diameter) and are devoid of TT [39]. In sinus venosus pacemaker cells of the cane toad, *Bufo marinus*, about two-thirds of the Ca^2+^ signal elicited by depolarization is owing to Ca^2+^ release from the SR. This CICR mechanism requires Ca^2+^ influx, largely via LTCCs and by NCX transporters operating in reverse mode [40]. These observations suggest that amphibian cardiac ECC resembles that of reptiles; the cardiomyocytes are narrow, lack TT and use both LTCC- and NCX-mediated Ca^2+^ influx to trigger CICR from the SR.

Of all teleost fishes (the largest infraclass of Actinopterygii, or ray-finned fish) studied, none are reported to possess TT or dyads in their cardiomyocytes. This has been found in many teleost species: in the myocardium of European plaice (*Pleuronectes platessa*) [35]; the ventricles of the rainbow trout (*Oncorhynchus mykiss*) [65]; the atria of three species of Gadidae (northeastern Atlantic cod, *Gadiculus thori*, fourbeard rockling, *Enchelyopus cimbrius*, and the haddock, *Melanogrammus aeglefinus*) [66]; the myocardia of pike (*Esox lucius*) and mackerel (*Scomber scombrus*) [27]; the atria and ventricles of the common carp (*Cyprinus carpio*) [28]; the ventricles of the African catfish (*Clarias gariepinus*) [29]; the ventricles of the albacore, or longfin tuna (*Thunnus alalunga*) and the Pacific bluefin tuna (*Thunnus orientalis*) [30,31]; and in ventricular myocytes of the zebrafish (*Da. rerio*) [32]. In several of these studies, both peripheral (subsarcolemmal) SR and extensive caveolae were noted, e.g. [27,31]. In a review of teleost cardiac ECC, it was suggested that lower density and greater distribution of RyR2 channels in the SR results in weaker CICR compared with that in endothermic vertebrates [33]. However, there is plasticity in the function of the SR in fishes with high-performance circulatory systems. For example, during cold-adaptation of the Pacific bluefin tuna, the volume and Ca^2+^-storage capacity of the SR is enhanced [34].

Among Chondrichthyes (cartilaginous fishes), ultrastructural investigation of elasmobranchs (clearnose skate (*Raja eglanteria*), the sandbar shark (*Carcharhinus plumbeus*) and the smooth dogfish (*Mustelus canis*)), did not detect the presence of TT in atrial or ventricular myocytes. In these species, inhibition of SR fluxes (by a combination of thapsigargin and ryanodine) decreased net force and increased relaxation time in strips of atrial and ventricular myocardium, suggesting a role of CICR [67].

The Atlantic hagfish *Myxine glutinosa* (order: Myxiniformes) has a cardiovascular system with six main contractile organs: a branchial (or main) heart, which transports blood from the gills to the body; a portal heart which conveys blood from the gut to the liver; two caudal hearts and two cardinal hearts. In this organism, peripheral SR couplings were found in both the portal heart and in the ventricle of the branchial heart, but no TT were reported [68]. The river lamprey (*Lampetra fluviatilis*) belongs to the order Petromyzontiformes, which have a well-developed heart, with a single atrium and ventricle as contractile components. Myocytes from the *La. fluviatilis* heart are long, but unlike most other ectothermic vertebrates, are relatively wide (a diameter of about 12 μm for ventricular myocytes). Ultrastructurally, lamprey cardiomyocytes contain an extensive SR, interacting with the sarcolemma at peripheral junctions, but TT were not observed. Functionally, cardiac ECC in *La. fluviatilis* depends on LTCC, with some force production requiring SR Ca^2+^ stores that are sensitive to ryanodine and caffeine (modulators of RyR gating) [26]. This SR Ca^2+^-release might serve to propagate Ca^2+^ signals in these relatively large cardiomyocytes, by CICR.

### (c) Invertebrates

In this section, the term ‘invertebrates’ refers to non-vertebrate metazoans. Studies on the ultrastructure of invertebrate heart contractile cells have been comprehensively reviewed [24], although not recently.

Deuterostomes are a superphylum of animals in which, during embryonic development, the blastopore (first opening) becomes the anus. This group includes chordates (vertebrates, cephalochordates and tunicates), hemichordates and echinoderms. Among tunicates, cardiomyocytes of the ascidiacean *Corella willmeriana* contain peripheral couplings, but no TT [25]. There are no reports on the cardiomyocyte SR ultrastructure of other chordate sister groups Cephalochordata (e.g. the lancelets, *Branchiostoma* spp*.*) and Hemichordatea (e.g. the acorn-worm, *Saccoglossus kowalevski*). The cardiovascular systems of echinoderms (starfish, brittle-stars, sea-urchins, sea-lilies and sea-cucumbers) are anatomically simple compared with other deuterostomes. The muscle cells of the sea-cucumber *Parastichopus tremulus* dorsal hemal vessel lack Z-discs and TT, but possess an SR system with peripheral couplings [13].

Together with the deuterostomes, protostomes comprise the majority of bilaterian animals. Protostomes were once defined as having the blastospore forming the mouth during development, but the embryogenesis of different taxa within this group is more diverse. The lophotrochozoans are a major superphylum of protostomes which includes molluscs, annelids, brachiopods, bryozoans and others. Most molluscan taxa possess an open circulation with a simple heart tube. Molluscan cardiomyocytes are variable in diameter (2–28 μm), depending on species and anatomical location. TT and dyads have been reported in cardiomyocytes of some molluscs, such as the snail, *Helix aspersa* [23] and the mussel *Mytilis edilis* [22]; but not in others such as the chitons *Lepidopleurus asellus* and *Tonicella marmorea*. No peripheral couplings between the SR and sarcolemmal were observed in these chitons [14]. Cardiomyocytes from the Busycon whelk (*Busycon canaliculatum*) are reported to lack typical TT, and have an extensive SR system, with peripheral couplings and interconnected extra-junctional segments deep within the myoplasm [15]. These were proposed to serve a similar Ca^2+^ signal propagating function to the corbular SR of bird and mammalian hearts. *Busycon canaliculatum* cardiomyocytes possess multiple shallow invaginations of the surface membrane, that were termed ‘sarcolemmal tubules’. Whether these sarcolemmal tubules are a rudimentary TT system, or if they represent caveolae, awaits further experimental investigation.

Like mammalian smooth muscle, the contractile cells of annelid hearts, or pseudohearts, are non-striated [16,24]. TT have not been reported in myocardia of this group, but they possess SR systems ranging in volume from sparse in *Siboglinum fiordicum* [17], to intermediate in the earthworm *Eisenia foetida* [18], to extensive in the lugworm *Arenicola marina* [19]. Peripheral couplings are present in all three species.

The ecdysozoans are a superphylum of protostomes that includes nematodes, priapulids, onychophorans, loriciferans, kinorhynchans, tardigrades and arthropods. Of these, only onychophorans are reported to possess cardiovascular systems. However, it should be noted that the body-walls of nematodes contain striated muscles, with TT that form dyad and triad junctions with the SR [24].

Dyad and triad SR junctions with the TT have been reported in the cardiomyocytes of most classes of arthropod, including the horseshoe crab *Limulus polyphemus* [20]; the Kuruma prawn *Penaeus japonicas* [21]; the common woodlouse *Oniscus asellus* and the pond slater *Asellus aquaticus* [5]; the orb-web spider *Nephila clavata* [6] and the fruit fly *Drosophila melanogaster* [7]. In addition to these TT-SR couplings, peripheral SR couplings have also been noted in cardiomyocytes from the amphipod *Tmetonyx cicada* [8]; the dragonfly *Sympetrum danae* [9]; and the tadpole shrimp *Lepidurus arcticus* [10].

The historical term Radiata refers to non-bilaterian animals. These include coelenterates: the cnidaria (jellyfish, sea-anemones and corals) and the ctenophores (comb-jellies). Although they lack hearts, coelenterates do possess smooth muscle cells. Two distinct populations of SR have been identified in the two types of giant smooth muscle fibres of the ctenophore *Beroe ovata*: it is homogenously distributed in the radial fibres but is located more centrally in the longitudinal fibres. Invaginations of the surface membrane were not observed [11]. Similarly, couplings between the sarcolemma and SR of the ctenophore *Mnemiopsis leydii* were not detected [12].

Other metazoan groups, such as the poriferans (sponges) and placozoans, do not possess smooth or striated muscle. Consequently, it is unlikely that these animals contain SR or TT, but their genomes encode precursors of the molecular machinery necessary to produce such membrane contact sites [51].

## 3. Molecular hallmarks of the cardiac dyad

Even in mammalian cardiomyocytes, the molecular machinery required for the formation of dyad junctions has not been completely elucidated. However, three proteins known to participate in dyad formation and function will be analysed in the current work: the bridging integrator 1 (BIN1), junctophilin 2 (JPH2) and caveolin 3. The evolution and roles of RyR2 and Ca_v_1.2 in heart dyad physiology have been extensively evaluated in previous publications [4,51], figure 1. Other proteins that potentially contribute to cardiac dyad formation and function include telephonin (titin-cap protein), mitsugumin 53 and nexilin. These have been comprehensively discussed in a recent review of the mammalian TT system [56].

### (a) The bridging integrator 1

BIN1 has several pseudonyms, including amphiphysin 2 and myc box-dependent-interacting protein 1. These names reflect the functional diversity of this protein: it is involved in both the generation and sensing of the curvature of biological membranes, in processes including endocytosis, synaptogenesis, tumour suppression, regulation of the actin cytoskeleton and TT formation. This pleiotropic functionality reflects structural diversity, resulting from alternate messenger RNA splicing of multiple exons. BIN1 is a member of the BAR (named after three proteins: BIN1, Amphiphysin and yeast Rvs167) domain, involved in dimerization to form a banana-shaped structure; for review, see [69]. Multiple homologues of BIN1 exist: these include orthologues in other species (genes derived by common ancestry whose products serve similar functions) and paralogues (genes derived by duplication whose products have diversified functions). The human genome encodes four homologues: the orthologue BIN1 (amphiphysin 2) plus three paralogues, namely BIN2, BIN3 and amphiphysin 2. A pre-computed gene gain/loss tree of homologues of *Homo sapiens* BIN1 is available at the Ensembl 2021 website, release version 105 (http://www.ensembl.org/Homo_sapiens/Gene/SpeciesTree?db=core;g=ENSG00000136717;r=2:127048027-127107288) [70]. This tree indicates complex patterns of loss and expansion of BIN1 homologues during evolutionary history. The yeast *Saccharomyces cerevisiae* possesses six homologues, whereas the ecdysozoans *Caenorhabditis elegans* and *Dr. melanogaster* have one. The tunicates *Ciona intestinalis* and *Ciona savignyi* have 2 BIN1 homologues, which on the basis of their protein sequence identities, are likely to be BIN1 orthologues. The hagfish *Eptatretus burgeri*, the lamprey, *Petromyzon marinus*, and the coelacanth, *Latimeria chalumnae*, all have three BIN1 homologues. Most of the vertebrates investigated possess four BIN1 homologues, but there is gene family expansion in teleost fishes, with many taxa encoding six homologues, salmonids between 9 and 11, and the goldfish *Carassius auratus*, having 15. There appear to be BIN gene losses and gains in other vertebrate groups: such as the blue-ringed sea krait, *Laticauda laticaudata*, having one homologue and the sloth, *Choloepus hoffmanni*, two; in contrast to the white-tufted-ear marmoset, *Callithrix jacchus*, which has seven. The consequences of such expansion or loss of BIN1 homologues on cardiac dyad function awaits characterization.

Early heterologous expression studies indicated that of isoforms of BIN1 present in mammalian skeletal muscle, those containing exon 11 promoted membrane infolding. This exon encodes a phosphoinositide-binding domain, located near the centre of the protein, which recruits BIN1 to the surface membrane where these lipids are enriched [71]. The role of BIN1 in the heart was first defined in transgenic mice, with a whole-body knockout of this gene. These mice died perinatally but displayed no apparent abnormalities in their skeletal muscle. BIN1 knockout embryos showed severe ventricular myopathy, with myofibrillar disorganization, indicating a role for this protein in heart development [72]. In mouse, rat and human cardiomyocytes, BIN1 facilitates microtubule-dependent trafficking of Ca_v_1.2 to cardiac TT, thereby enhancing ECC. This effect was dependent of the C-terminal 172 amino acid residues of BIN1 and not on its N-terminal BAR domain [73]. In cardiomyocytes from failing human hearts, BIN1 protein level, TT density and Ca_v_1.2 trafficking are reduced [74]. In mouse cardiomyocytes, the β-adrenoreceptor agonist isoproterenol stimulated phosphorylation of RyR2, promoting its interaction with BIN1 and recruitment to dyads. In rat ventricular cardiomyocytes, silencing of BIN1 expression using short interfering RNA reduced TT density and enhanced the heterogeneity of systolic Ca^2+^ transients [75]. The major form of BIN1 in mouse cardiomyocytes, BIN1 + 13 + 17 (containing exons 13 and 17), promoted N-Wiskott–Aldrich syndrome protein (N-WASP)-dependent actin polymerization, which stabilized TT at the Z-discs [76]. In human stem cell-derived cardiomyocytes, increases in BIN1 abundance were associated with TT nucleation, growth and RyR2–Ca_v_1.2 interactions at CRUs. In contrast to an earlier study, it was found that all five spice-variants of BIN1 present in rat could generate functional TT, with those containing the PI-binding motif being particularly effective at this activity [77].

There is less information about the role of BIN1 in non-mammalian hearts. In the zebrafish heart, morpholino-knockdown of BIN1 decreased the amplitude of Ca^2+^ transients and severely compromised ventricular contractility [74]. *Drosophila melanogaster* BIN1 plays a key role in the development of TT in body muscle and is also detected at the protein level in the heart of this fruit fly [78]. A homologue of BIN1 has also been isolated from the sponge *Geodia cydonium*. This BIN1 homologue does not appear to be involved in the organization of subcellular structures, as it is a secreted aggregation factor involved in colony formation by this sponge [79].

**Figure 2.**
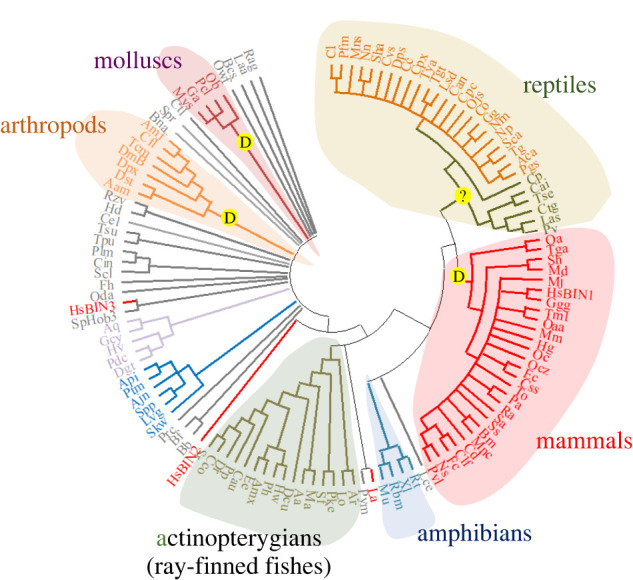
Phylogeny of BIN proteins in metazoans. The evolutionary history of BIN1 and homologous proteins was inferred using the maximum likelihood with the Jones–Taylor–Thornton substitution matrix, essentially as described previously [55]. The tree shown represents a consensus of 500 bootstrap replicates; branches were collapsed in cases where they were present in less than 50% of these replicates. This analysis was based on 124 protein sequences, obtained using BLAST searches of *Homo sapiens* BIN1 isoform 1 (Accession Number NP_647593.1) and selecting representative taxa from the hits obtained (for full details including the key to species name abbreviations, see the electronic supplementary material, table S1). Outliers include *H. sapiens* BIN2 and 3 (HsBIN2, HsBIN3), a *Rhodamnia argentea* (green plant) BIN homologue (Rag), *Schizosaccharomyces pombe* SpHob3 (yeast homologue of BIN3), and a candidate BIN homologue from the choanoflagellate *Salpingoeca rosetta* (Spr). Taxa in which there is ultrastructural evidence of dyads in cardiomyocytes are indicated with (D); where this evidence is contentious, it is indicated by (?).

BIN homologues are represented throughout Eukarya, including in yeast, green plants and animals (see the electronic supplementary material, table S1). In the current study, phylogenetic reconstruction of the evolution of BIN1 (using the MEGA X package, [80]) indicates that this BAR protein family member probably evolved in vertebrates, figure 2. The presence of cardiac TT and dyads in different taxonomic groups indicates that these membrane junctions have evolved independently, at least twice: once or more in protostomes (including ecdysozoans and lophothrocozoans), and more recently in mammals (or alternatively, in a tetrapod common ancestor of mammals and reptiles).

**Figure 3.**
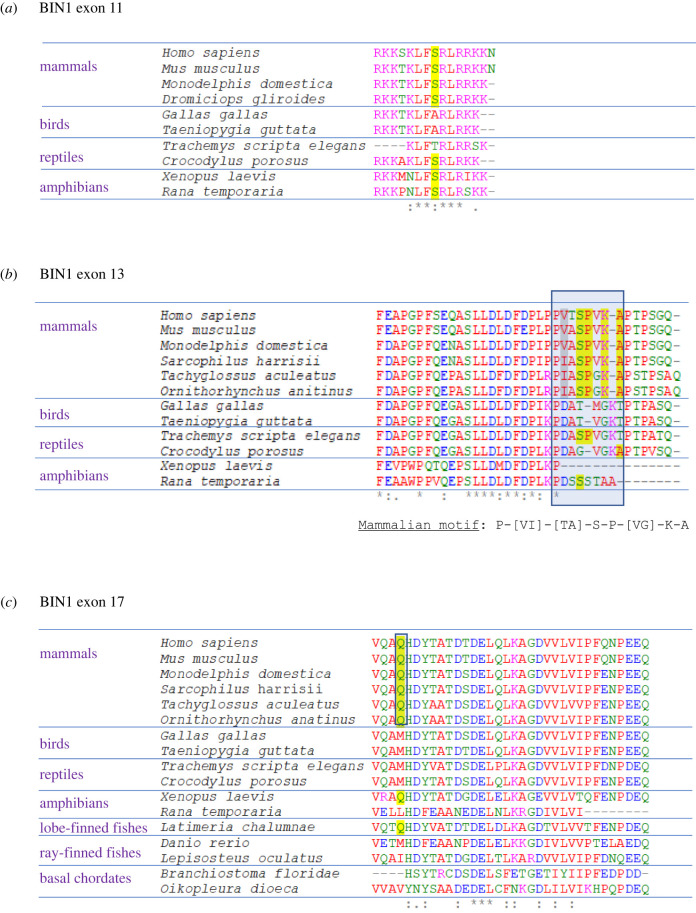
Multiple sequence alignments of peptides encoded by BIN1 exons 11, 13 or 17. These peptides are reported to play roles in TT formation in striated muscles. In all cases, the human homologues of these peptides were retrieved by BLAST searching against the PubMed Protein database, essentially as described previously [55]. (*a*) shows the alignment of exon 11 (with the query sequence derived from *H. sapiens* BIN1 + 11 + 17 (acc. no. NP_647596.1)); (*b*) that of exon 13 (from *H. sapiens* BIN1 + 13 + 17 (acc. no. NP_647593.1)), with a motif conserved in mammals but not in other vertebrates indicated in a blue shaded box; and (*c*) for exon 17 (from *H. sapiens* BIN1 + 13 + 17 (acc. no. NP_647593.1)). Beneath each alignment, the symbol ‘*’, indicates complete conservation of that residue; ‘:’, the presence of a homologous substitution; and ‘.’, a partially homologous substitution. Residues that are conserved between mammalian taxa but not in other vertebrates are highlighted in yellow.

The role of BIN in the evolution of cardiac dyad junctions is uncertain. The BIN homologues present in molluscs (lophotrochozoans) and arthropods (ecdysozoans) do not group with vertebrate BIN1 in phylogenetic reconstructions (figure 2). It is unclear if these homologues represent orthologues or paralogues of human BIN1, since they share low sequence identity (30–40%) with this protein. To gain insights into features of vertebrate BIN1 that might be required for TT production, multiple sequence alignments (using the Clustal-Omega package, [81]) of three exons potentially involved in this process were performed using sequences from mammals which have detectable cardiac dyads and other vertebrate taxa, such as birds, which do not. Of these, homologues of the sequence encoded by *H. sapiens* BIN1 exon 11 were not detectable in monotremes (egg-laying mammals), a tetrapod (the coelacanth, *Latimeria chalumnae*, a lobe-finned fish), ray-finned fishes or invertebrates. The only amino acid conserved in mammals but not birds are a serine residue; figure 3*a*. Analysis of this site using the NetPhos algorithm (https://services.healthtech.dtu.dk/service.php?NetPhos-3.1) [82] indicates that it is potentially phosphorylated by cyclic AMP-dependent protein kinase (PKA consensus site score of 0.7, threshold 0.5). However, it is not known if phosphorylation of BIN1 by PKA, or any other kinase, regulates its function. Furthermore, this serine is also conserved in amphibians and some reptiles, indicating that it might not confer mammal-specific functions.

Homologues of *H. sapiens* BIN1 exon 13 were only detected in mammals, birds, reptiles, amphibians, lobe-finned fishes, some sharks and a limited range of ray-finned fishes. Comparison of aligned sequences revealed a motif that was conserved in mammalian BIN1 homologues, but not in those from non-mammalian species, figure 3*b*. Searching for this motif in the ProSite database, using the ScanProsite tool [83], revealed that it is present in mammalian BIN1 homologues and in complex I intermediate-associated protein 30 (CIA30). This protein is a molecular chaperone, responsible for the initial stages in assembly of complex I of the mitochondrial electron transport chain [84]. It is tempting to speculate that this motif is also involved in certain protein–protein interactions mediated by BIN1.

Homologues of human BIN1 exon 17-encoded peptide are detectable in all vertebrate classes and in other chordates, but only in some echinoderms, lophotrochozoans, ecdysozoans and cnidarians, figure 3*c*; electronic supplementary material, table S1. Comparison of human BIN1 with those from other taxa indicate that there is a glutamine residue (Q529 in isoform 1) encoded by exon 17 that is conserved in mammals, but not in non-mammalian animals. This residue might underpin the difference between mammals and other vertebrates in the presence of cardiac dyads: a hypothesis that is testable using mutation and heterologous expression approaches.

### (b) Junctophilins

JPH are a family of proteins that link intracellular organelles, like the SR, to surface membranes such as the sarcolemma and TT [85]. This is achieved through a transmembrane domain in the organelle, a helical spanning (or joining) region and an N-terminal membrane organization recognition nexus motif, which binds to anionic lipids in the inner leaflet on surface membranes. Invertebrates and choanoflagellates (a sister taxon of metazoans) encode a single JPH homologue, whereas most vertebrate genomes encode four (except for birds, which have three members) [51]. In mammals, JPH1 is found at highest levels in skeletal muscle, whereas JPH2 is present in both types of striated muscle. Transgenic mice lacking a functional JPH2 gene die mid-gestationally, showing heart tube defects, and an expansion of the dyadic clefts, along with a reduction in their number [85]. These observations indicate that JPH2 is essential for the formation of CRUs in the heart, at both dyads and peripheral couplings. In addition to this tethering role, JPH2 also facilitates communication between RyR2 and Ca_v_1.2 at these CRUs [86]. Recently, it was demonstrated that the joining region of JPH2 interacts directly with Ca_v_1.2, recruiting the LTCC to the cardiac dyad [87]. In invertebrate cardiovascular systems, insights into JPH physiology were developed through genetic manipulation of the arthropod, *Dr.melanogaster*. Heart-specific silencing or overexpression of the single JPH gene in this fruit fly significantly decreased lifespan and was associated with enlargement of the heart tube, with disorganization of the contractile apparatus [88].

### (c) Caveolin 3

Caveolae are flask-shaped infoldings of cell-surface membranes, which are nucleation sites for the production of TT in striated muscles [56]. Caveolins (CAV) are integral membrane proteins that are key components of the caveolae, recruiting distinctive populations of proteins and lipids to these structures. Phylogenetic analyses indicate that CAVs arose early in metazoan history and diverged into two main groups: those that form caveolae and those which do not [89]. In mammals, CAV3 is restricted to smooth and striated muscle cells. CAV3 knockout mice display cardiac hypertrophy, decreased LTCC current density (in TTs) and altered TT structure [90]. Conversely, cardiomyocyte overexpression of CAV3 in transgenic mice abrogates loss of TT LTCC current density in a transverse aortic restriction model of heart failure [90]. CAV3 also interacts with JPH2, potentially serving as an anchoring point for the SR during the formation of peripherally and dyadic CRUs in cardiomyocytes [3]. In terms of invertebrate homologues of this family, heterologous expression of a honeybee (*Apis mellifera*) CAV in a mammalian cell-line resulted in *de novo* formation of caveolae [89]. This demonstrates that caveolae formation is a conserved property of this lineage of CAVs, and that these could contribute to the formation of TT and dyads observed in arthropod cardiomyocytes.

## 4. Conclusion and perspectives

The current work provides insights into the evolutionary history of cardiac dyads and the roles of key proteins in their formation. A fundamental perspective is that these junctions developed independently several times during metazoan evolution, representing homoplasy (convergent evolution). Ultrastructural evidence unambiguously supports the presence of TT and dyads in the cardiomyocytes of mammals, arthropods and some molluscs. A point of conflict is whether the myocardium of reptiles, in particular turtles, possess ‘short-TT’ coupled to the SR. This is of importance and is worthy of further experimental investigation, as it places the evolution of vertebrate cardiac dyads either to a common ancestor of mammals and reptiles, or to the point of divergence of mammals from other vertebrate groups. If the former scenario is correct, it implies that birds must have subsequently lost the ability to generate cardiac dyads. This is not unreasonable, as birds are known to have lost members of protein families involved in ECC and Ca^2+^ signalling, such as JPH4 [51]. It is also important to highlight that triad junctions in skeletal muscle are distinct entities from dyad junctions in the heart. In mammals, these junction types possess different populations of proteins (e.g. RyR1/Ca_v_1.1/JPH1 in skeletal muscle and RyR2/Ca_v_1.2/JPH2 in cardiac muscle) and possibly have distinct mechanisms of formation (e.g. dependent on BIN1 + 11 versus BIN1 + 13 + 17). These membrane junctions also show distinct patterns of emergence during animal evolution, e.g. ray-finned fishes possess triad junctions in their skeletal muscles but lack dyad junctions in their hearts.

Comparison of the phylogenetic and molecular features of proteins involved in dyad formation also supports a homoplastic mechanism of evolution. For example, BIN homologues from arthropods and molluscs do not group with vertebrate BIN1 proteins in phylogenetic analyses, nor do they share conserved exons involved in TT formation. Multiple sequence alignments of the peptides encoded by these exons revealed a glutamine residue in exon 17 (Q529) that is conserved in mammals (which form TT) but not in other vertebrates (which lack these structures). This residue potentially has a function in the formation of cardiac dyads in mammals.

Based on the ultrastructural, transcriptomic, phylogenetic and physiological analyses of the different muscle types present in the marine annelid *Platynereis dumerilii*, Brunet *et al*. developed a framework for the evolutionary origin of smooth and striated myocytes in bilaterians [92]. This model proposed the existence of body-wall striated muscle and visceral smooth muscle in a deuterostome–protostome common ancestor. In some later-branching organisms, populations of visceral smooth myocytes co-opted striated muscle features to develop into cardiomyocytes. This framework is useful in the interpretation of the ultrastructural, physiological and molecular findings related to cardiac dyad junctions summarized in the current work. Cardiomyocytes from early-diverging bilaterians share many features with smooth muscle cells and may resemble an ancestral, intermediate state in the transition between these two muscle types: they are narrow and spindle-shaped; rely mainly on Ca^2+^ influx through LTCC to drive ECC; and use the SR as a reserve mechanism in situations demanding high cardiac output [33]. The independent development of cardiac dyads at several points during evolution is unexpected and suggests advantages of these subcellular junctions under certain circumstances. Under these circumstances, cardiac dyads could have evolved by modification of pre-existing components, such as BINs, CAVs, JPHs, RyRs and LTCCs [51]. This is analogous to proposed mechanisms for the evolution of the actin–myosin contractile apparatus in metazoans: addition of new components to pre-existing ones facilitated the rapid development of this complex trait [93].

## Data Availability

The complete dataset used in this manuscript is available in electronic supplementary material, table S1 and in the main body of the text. The data are provided in the electronic supplementary material [94].
